# Feasibility of Ambient Home Sensing Technology for People Requiring In‐Home Professional Care

**DOI:** 10.1002/alz70858_101297

**Published:** 2025-12-25

**Authors:** Steven Ferguson, Shadi Gholizadeh, Timothy Thomas, Joey Taylor, Derek Gordon, Nathanial Findlay

**Affiliations:** ^1^ Lifeguard Health Inc., Québec, QC, Canada; ^2^ TheKey (Formerly Home Care Assistance), San Diego, CA, USA; ^3^ TheKey Research Group, San Diego, CA, USA; ^4^ TheKey, Montreal, QC, Canada; ^5^ TheKey, Irvine, CA, USA

## Abstract

**Background:**

Innovative technologies such as ambient home sensors have the potential to transform dementia care by offering non‐invasive, low‐cost, and easy‐to‐install solutions. These systems provide actionable insights into critical aspects of daily living, such as nutrition, hygiene, sleep, activity, and medication adherence. This feasibility study aimed to assess the openness of families to integrating this technology into home care practices.

**Method:**

A feasibility study was conducted with 25 participants across 23 homes who had professional home care for at least one month. Client care managers (*N* = 8) provided feedback from five cities: Montreal, Toronto, Winnipeg, Calgary, and Vancouver. Two modes of ambient sensing technology were utilized: one focused on falls and the other on broader monitoring capabilities, including wandering and inactivity detection. Both systems sent notifications to an alert center. Participants were introduced to three proposed use cases for the sensors:

**1. Caregiver Oversight Only**: Providing insights into professional caregiver behavior and engagement patterns.

**2. Person Oversight Only**: Delivering person‐specific insights to supplement or replace in‐home care.

**3. Dual Oversight**: Offering combined insights for both caregivers and people living with cognitive changes.

Qualitative feedback was collected through unstructured interviews and assessments with families and client care managers to evaluate preferences, perceived benefits, and potential barriers to adoption.

**Result:**

The dual oversight model was preferred by most participants due to its ability to enhance safety and provide peace of mind. The caregiver oversight‐only model was valued in scenarios requiring accountability for professional care, while the client oversight‐only model was favored as a standalone monitoring solution. Privacy concerns and the learning curve associated with app usage were identified as key barriers to adoption. Participants also highlighted the importance of customization to meet individual care needs.

**Conclusion:**

Ambient home sensors are a feasible addition to dementia care practices, with broad support for the dual oversight model among families. Tailored implementation strategies addressing privacy and usability concerns will be crucial for successful adoption. Future research should explore the long‐term impact of these technologies on care outcomes and family satisfaction.

